# Mésothéliome malin de l´ovaire: à propos d´un cas

**DOI:** 10.11604/pamj.2021.38.92.24462

**Published:** 2021-01-27

**Authors:** Imane Ouafki, Lamyae Nouiakh, Raihana Boujarnija, Afaf Amarti, Lamiae Amaadour, Karima Oualla, Zineb Benbrahim, Samia Arifi, Nawfel Mellas

**Affiliations:** 1 Service d´Oncologie Médicale, Centre Hospitalier Universitaire (CHU) Hassan II, Fès, Maroc,; 2 Centre d´Anatomo-pathologie Al Azhar, Fès, Maroc

**Keywords:** Mésothéliome, ovaire, primitif, chimiothérapie, à propos d´un cas, Mesothelioma, ovary, primitive, chemotherapy, case report

## Abstract

Le mésothéliome malin primitif de l´ovaire (MMPO) est une tumeur extrêmement rare qui peut se développer à partir des cellules mésothéliales. Cette néoplasie est causée principalement par une exposition à l´amiante ou à d´autres agents cancérigènes. Un bilan d´extension préopératoire comportant une tomodensitométrie, une imagerie par résonance magnétique et une tomographie par émission de positons est essentiel pour la stadification de la maladie. Le diagnostic positif anatomopathologique repose sur un panel immunohistochimique. Le MMPO reste une maladie exceptionnelle impliquant une stratégie thérapeutique multidisciplinaire, au sein de laquelle la chimiothérapie a permis d´améliorer la prise en charge et le pronostic de ces malades. Nous présentons dans cet article le cas d´une patiente ayant subi une chirurgie suboptimale, complétée par une chimiothérapie adjuvante, aboutissant à une réponse complète radiologique, avec une survie sans maladie de plus d´une année.

## Abstract (en)

Primary malignant mesothelioma of the ovary (PMMO) is an extremely rare tumor which can develop from mesothelial cells. This neoplasia is caused predominantly by exposure to asbestos or other cancer-causing agents. Preoperative assessment, based on computed tomography (CT) scan, magnetic resonance imaging and positron emission tomography, is essential for cancer staging. Anatomopathological diagnosis is based on immunohistochemical findings. PMMO is an exceptional disease involving a multidisciplinary therapeutic strategy including the use of chemotherapy which improves the management and prognosis of patients. This study reports the case of a female patient undergoing suboptimal surgery complemented by adjuvant chemotherapy with complete radiological response and 1-year disease-free survival.

## Introduction

Le mésothéliome malin (MM) est une néoplasie agressive rare mais rapidement mortelle, qui affecte les cellules du mésothélium, membrane protectrice qui recouvre la plupart des organes internes du corps. On le retrouve notamment au niveau: des poumons (la plèvre 85,5%), du cœur (le péricarde), des intestins (le péritoine 13,2%), des testicules, de la tunique vaginale et des ovaires. Il a d´abord été décrit par Miler et Wyn en 1908 [1, 2]. La période de latence du MM est exceptionnellement longue et les patients diagnostiqués sont généralement de mauvais pronostics, avec une espérance de vie moyenne de moins d´un an, en fonction du tissu dont il est originaire [3]. En effet, le diagnostic précoce de la maladie est difficile, retardant ainsi un traitement efficace. Il a été rapporté que l´amiante est le principal carcinogène associé au MM [3]. Cependant, le mécanisme moléculaire responsable de la pathogénèse du MM n´a pas été élucidé. Bien que l´atteinte de l´ovaire peut être observée au cours d´un MM péritonéal diffus, la présentation primitive par une tumeur ovarienne est très inhabituelle [2]. Nous rapportons ici un cas de MMPO. Ses caractéristiques cliniques, les résultats radiologiques, histopathologiques détaillés, de même que la prise en charge thérapeutique seront discutés.

## Patient et observation

**Figure 1:**
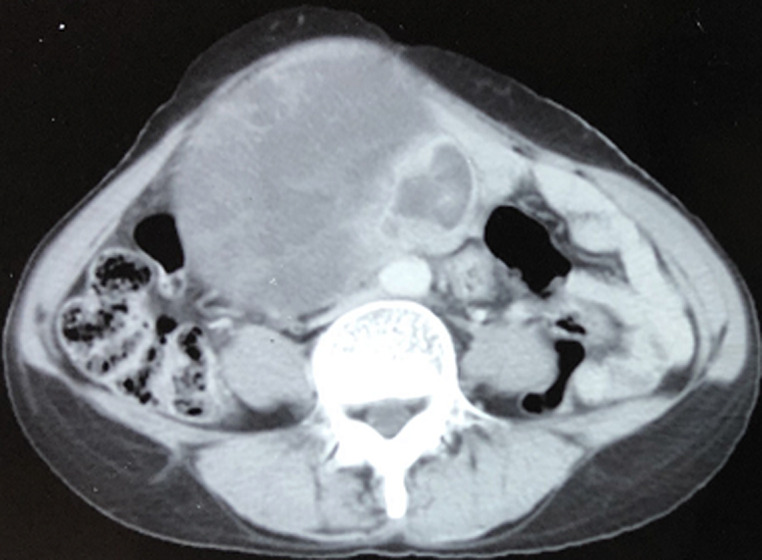
aspect au scanner thoraco-abdomino-pelvien objectivant une volumineuse masse latéro et sus utérine gauche grossièrement ovalaire bien limitée hétérogène à double composante kystique majoritaire contenant des septas épaissies et tissulaire, rehaussée de façon hétérogène après contraste, mesurant 145 mm x 100 mm x 123 mm

Il s´agit d´une patiente de 50 ans, grande multipare, ayant comme antécédents pathologiques une insuffisance cardiaque gauche sur une cardiopathie ischémique stable sous anti-vitamine K et Aspirine, hypertendue bien équilibrée sous traitement anti-hypertenseur et mesures hygiéno-diététiques. Son histoire de la maladie avait commencé par son hospitalisation dans le service de gynécologie pour la prise en charge d´une masse abdomino-pelvienne arrivant jusqu´à l´ombilic faisant 20 cm de grand axe. Une échographie pelvienne réalisée parlait d´un utérus globuleux mesurant 74 mm x 41 mm, l´endomètre était régulier épaissi de 14 mm, une image hétérogène prenait tout l´écran, solido-kystique et hypoéchogène multiloculaire (plus de 6) avec des cloisons dont l´épaisseur est de 3 mm avec vascularisation centrale, en faveur d´une masse solido-kystique suspecte ovarienne. Le scanner thoraco-abdomino-pelvien avait objectivé une volumineuse masse latéro et sus utérine gauche grossièrement ovalaire bien limitée hétérogène à double composante kystique majoritaire contenant des septas épaissies et tissulaire, rehaussée de façon hétérogène après contraste, mesurant 145 mm x 100 mm x 123 mm (Figure 1).

L´hystéroscopie diagnostique avait montré une atrophie endométriale diffuse avec hypertrophie localisée au niveau de la face postérieure sans signes d´atypies, les deux ostiums et l´endocol était sans anomalies. Le curetage n´a pas été fait vue la non disponibilité de la pipelle de Cornier dans le service. Pour les marqueurs tumoraux le CA125=233 U/ml, l´ACE et le CA19-9 étaient normaux. L´inhibine et l’hormone antimüllérienne (AMH) n´ont pas été dosés par manque de réactifs. La laparotomie exploratrice réalisée avait trouvé une ascite de faible abondance prélevée pour cytologie avec découverte d´une masse faisant 30 cm x 40 cm à paroi lisse friable, hypervascularisée à composante hémorragique intra-kystique au dépend de l´ovaire gauche adhérente au sigmoïde du côté gauche, à noter la rupture per-opératoire accidentelle de la portion hémorragique de la masse qui fut protégée par des champs stériles.

**Figure 2:**
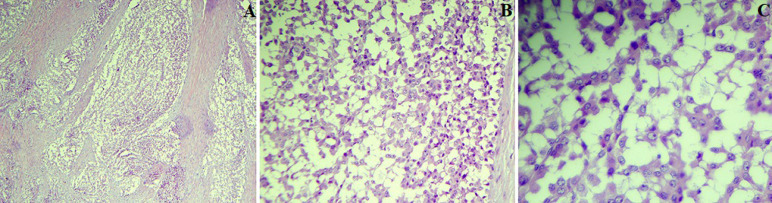
A) aspect microscopique après coloration à l’hématoxyline et éosine x40 (faible grossissement); B) aspect microscopique après coloration à l’hématoxyline et éosine x200 (moyen grossissement); C) aspect microscopique après coloration à l’hématoxyline et éosine x400 (fort grossissement)

Le reste de l´exploration chirurgicale avait objectivé un utérus de taille normale, une trompe gauche et une annexe droite sans anomalies, pas de carcinose péritonéale visible, le foie et l´estomac étaient lisses et il n´y avait pas d´adénopathies palpables. Une annexectomie gauche a été réalisée puis adressée pour un examen extemporané. Ce dernier parlait d´un processus tumoral malin, indifférencié. Puis le geste a été complété par une hystérectomie totale avec une annexectomie droite et des biopsies multiples. Le résultat de l´étude anatomopathologique était en faveur d´un processus carcinomateux avec un liquide péritonéal tumoral. A l´histologie: les différents prélèvements réalisés au niveau de la masse tumorale sus-décrite correspondent à une prolifération tumorale disposée en nappe diffuse, avec parfois des papilles, les cellules tumorales sont bordées par des cellules atypiques de grande taille, munies de noyaux arrondis, à chromatine fine, parfois nucléolés, et entourés d´un cytoplasme abondant éosinophile, avec présence de foyers de nécrose tumorale, des remaniements hémorragiques sont notés (Figure 2).

**Figure 3:**
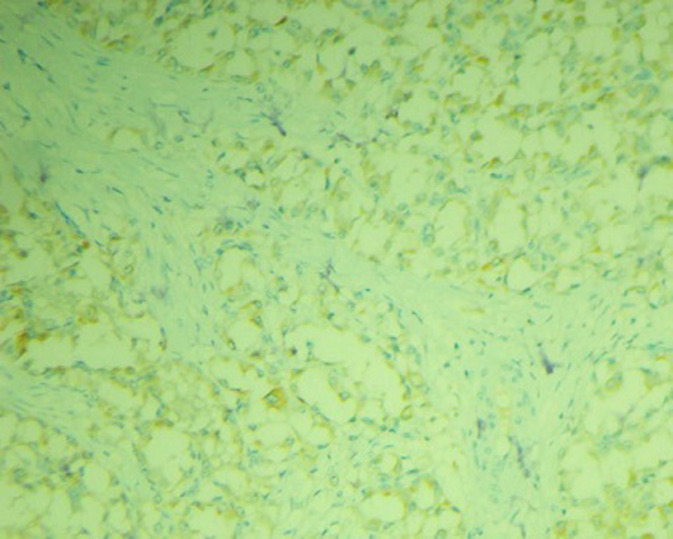
aspect immunohistochimique exprimant la faible positivité du Ber-EP4

**Figure 4:**
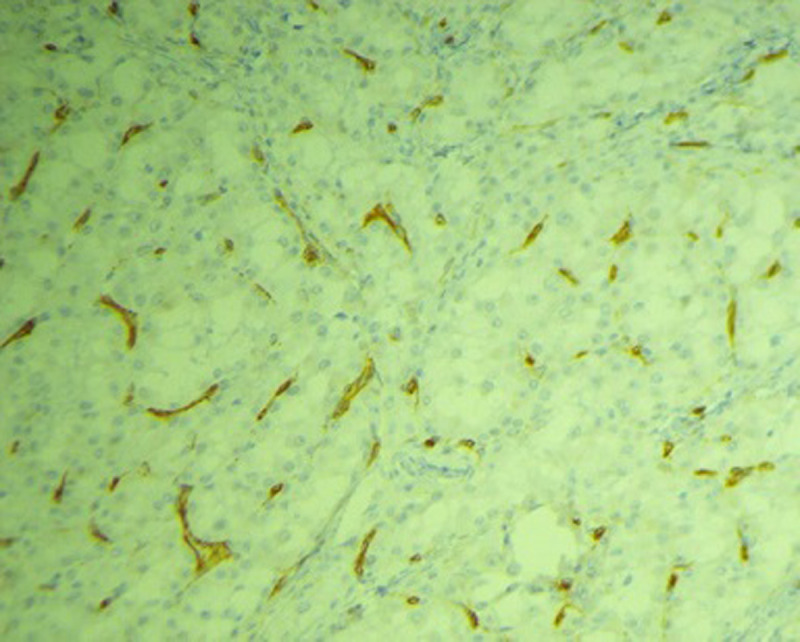
aspect immunohistochimique exprimant la négativité du WT1

**Figure 5:**
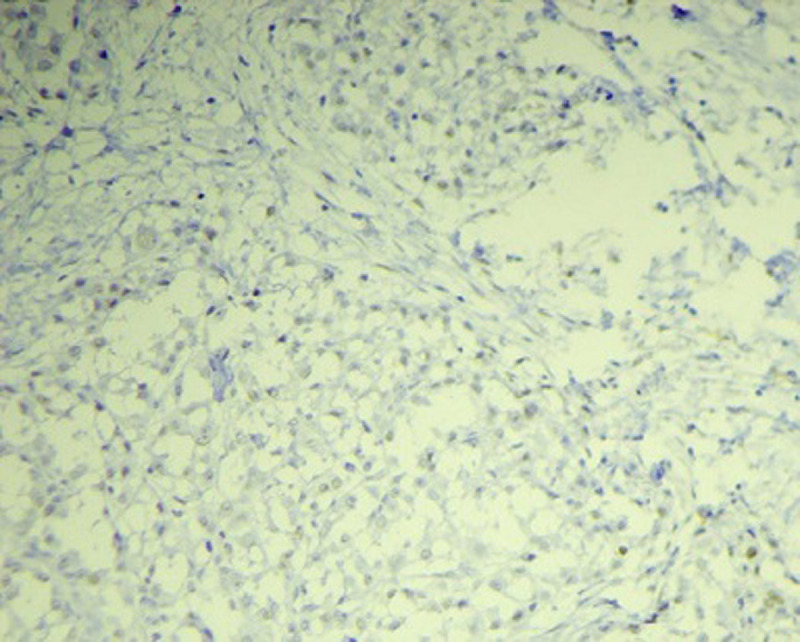
aspect immunohistochimique exprimant la négativité du TFE3

A l´immunohistochimie: les cellules tumorales expriment la CK7, la calrétinine, focalement l´inhibine, la vimentine, la P53, la CK8/18, la CK19 et la CK20. Elles n´expriment pas le CD30, la PS100, le TTF1, la thyroglobuline, la chromogranine, la synaptophysine, le CD56, la melan A, l´EMA ni la CK5/6. Ce panel oriente vers un mésothéliome ovarien. Un complément d´immunomarquage par le Ber-EP4 a été effectué qui était faiblement positif (Figure 3), en faveur d´un mésothéliome. Nous avons complété également par le WT1 (Figure 4) qui est normalement positif dans les carcinomes ovariens, qui est négatif dans notre cas. On a aussi réalisé le TFE3 qui est exprimé par les sarcomes alvéolaires vue la ressemblance morphologique et il était également négatif (Figure 5). L´index de prolifération tumorale évalué par le Ki 67 était estimé à 30%. Au final, le diagnostic de mésothéliome ovarien a été retenu. Le scanner post-opératoire n´avait pas montré de maladie résiduelle et le CA125 est passé à 21.40 U/ml. Sur ces données, la décision était de faire une chimiothérapie adjuvante. Le bilan biologique pré chimiothérapie était correct.

L´échographie cardiaque trans-thoracique avait mis en évidence une insuffisance tricuspidienne et mitrale minimes, un ventricule gauche de taille normale et de fraction d´éjection conservée à 62%. La patiente a été mise sous le protocole de chimiothérapie comportant la Gemcitabine (à j1 et j8) et le Carboplatine AUC 5 (à j1) tous les 21 jours (j1=j21). Le Pemetrexed n´était pas disponible dans notre structure au moment du premier cycle. La patiente avait reçu 6 cures de chimiothérapie, la tolérance était marquée par la survenue d´un épisode de neutropénie non fébrile qu´on a pu gérer par le repos et les mesures hygiéno-diététiques. Le scanner thoraco-abdomino-pelvien d´évaluation après 3 cycles avait confirmé l´absence de masse pelvienne ou de prise de contraste pathologique notamment au niveau de la tranche de section vaginale. Le même constat était retrouvé lors des contrôles scannographiques ultérieures. Actuellement, la patiente est en bon contrôle avec un recul de plus de 12 mois.

## Discussion

Le MM est une tumeur dérivée des cellules mésothéliales. L´exposition à l´amiante en est la cause la plus fréquente [1]. Cependant, plusieurs cas dans la littérature n´étaient pas associés à cette notion [1]. Notre patiente avait des antécédents négatifs d´exposition professionnelle à l´amiante, dans la mesure où elle est femme au foyer. Le MM peut survenir après une radiothérapie, ce qui suggère que l´irradiation directe pourrait être un facteur de risque pour son développement [1-4]. Dans le cas présent, il n´existe aucune notion de radiothérapie. D´autres facteurs ont été incriminés comprenant certains minéraux, tels que l´érionite, le thorium et le mica [1-5]. Néanmoins, la causalité liée à ces substances n´a été rapporté que dans des cas isolés et par conséquent le risque relatif de développer un MM n´a pas encore été quantifié [1].

Les mésothéliomes ovariens primitifs représentent environ 0,03% des décès liés au mésothéliome [6]. La localisation ovarienne est fréquente lorsqu´il y´a une atteinte diffuse ou multifocale du péritoine. Par ailleurs, la littérature n´a rapporté que quelques cas de MM se présentant principalement avec une masse ovarienne [2]. Goldblum et Hart avaient trouvé une maladie ovarienne secondaire dans 10 cas au cours de mésothéliomes péritonéaux diffus; l´âge moyen de la cohorte était de 52 ans [7]. Clement *et al*. avaient rapporté une série de 9 cas de MM se présentant sous forme de masses ovariennes. L´âge moyen des patientes était de 52 ans; 7 des 9 présentaient une tumeur péritonéale extra-ovarienne étendue, tandis que les tumeurs étaient confinées aux ovaires chez 2 patientes [8]. Dans sa série de 7 cas de mésothéliome, Merino avait des patientes entre 22 et 52 ans avec une moyenne d´âge de 32 ans [6]. Notre patiente est âgée de 50 ans, ce qui rejoint la littérature. Les premiers symptômes les plus fréquemment rapportés sont les douleurs abdominales (35%), avec une distension abdominale (31%), une anorexie, une perte de poids et une ascite [2].

Parfois, le diagnostic est posé fortuitement au cours d´une laparoscopie [2]. En immunohistochimie, le mésothéliome malin se caractérise par la positivité de la calrétinine, la cytokératine 5/6, l´EMA (Epithelial Membrane Antigen), le Wt1 (Wilms´ tumor gene 1), l´anticorps cellulaire anti-mésothéliale 1 et la mésothéline. Par ailleurs on note la négativité des marqueurs de tumeurs malignes du tractus gastro-intestinal y compris ACE et MOC-31 (ou B72.3, Ber-EP4 ou BG-8) [2]. Pour notre patiente, l´immunohistochimie a montré une positivité pour la cytokératine 7, la calrétinine, la vimentine, tandis qu´on a la négativité de la cytokératine 5/6, de l´EMA, et du Wt1. Le Ber-EP4 était faiblement positif. L´adénocarcinome et le mésothéliome malin ovariens co-expriment tous les deux la cytokératine, la vimentine et souvent l´EMA. Dans un autre travail, seuls Ber-EP4, MOC-31, le récepteur aux œstrogènes et la calrétinine ont été retenus comme marqueurs utiles pour ce diagnostic différentiel [2]. Dans l´ensemble, il est recommandé d´utiliser au moins deux marqueurs de mésothéliome et deux marqueurs de carcinome [9]. Dans l´ovaire, les mésothéliomes doivent être différenciés des autres néoplasies, notamment les lésions bénignes telles que le lymphangiome kystique, les formes kystiques d´endosalpingiose, l´endométriose et les tumeurs adénomatoïdes kystiques [6].

La stratégie thérapeutique repose sur une approche locorégionale par une chirurgie de cytoréduction optimale ou sous-optimale, qui augmente les chances de survie à long terme chez ces patientes. Une chimiothérapie systémique de première intention est raisonnable pour les tumeurs non résécables d´emblée, mais la réponse reste limitée. Cette chimiothérapie trouve sa place également en situation adjuvante en présence de facteurs de mauvais pronostic. Les drogues actives ayant été utilisées dans les différents cas rapportés sont: les sels de platine (Cisplatine, Carboplatine), la Gemcitabine et le Pemetrexed. Les protocoles de chimiothérapie administrés historiquement étaient l´association Irinotécan plus Carboplatine, idéalement la combinaison Cisplatine 50 mg/m^2^ plus Pemetrexed 500 mg/m^2^. La chimiothérapie intra-péritonéale avec divers agents dont le Cisplatine, la Mitomycine C, la Doxorubicine, l´Epirubicine, l´Etoposide et la Cytarabine, utilisés seuls ou en association a été également testée [2].

## Conclusion

L´atteinte primitive des ovaires par un mésothéliome malin est exceptionnelle et seuls quelques cas ont été rapportés dans la littérature. La différenciation se fait avec un mésothéliome péritonéal diffus, un carcinome ovarien et un carcinome péritonéal. De nouveaux marqueurs immunohistochimiques comme la calrétinine confirment le diagnostic. La prise en charge thérapeutique est pluridisciplinaire basée sur la chirurgie, la chimiothérapie et la radiothérapie, mais des stratégies plus efficaces sont nécessaires afin d´améliorer les résultats des approches conventionnelles. D´autres études sont requises pour un diagnostic précoce et correct. La connaissance de cette présentation est importante afin d´établir des thérapies chirurgicales et adjuvantes appropriées.
